# An unusual case of nephrotic syndrome

**DOI:** 10.1007/s00467-024-06408-3

**Published:** 2024-05-23

**Authors:** Dermot Michael Wildes, Aisling Fitzsimons, Brendan Doyle, Andrew Green, Clodagh Sweeney, Atif Awan

**Affiliations:** 1 RCSI University of Medicine and Health Sciences, 123 St. Stephen’s Green, Dublin 2, Ireland; 2 Department of Paediatric Nephrology and Transplantation, Children’s Health Ireland, Dublin, Ireland; 3 Department of Histopathology, Beaumont Hospital, Dublin 9, Ireland; 4 Department of Clinical Genetics, Children’s Health Ireland, Dublin, Ireland; 5 Department of Paediatrics, School of Medicine and Medical Science, University College Dublin, Belfield, Dublin 4, Ireland; 6 Department of Medicine, School of Medicine and Medical Science, University College Dublin, Belfield, Dublin 4, Ireland

**Keywords:** Alport syndrome, X-linked Alport syndrome, Nephrotic syndrome, Alport syndrome in women

## Abstract

**Background:**

Alport syndrome is a genetically heterogenous disorder resulting from variants in genes coding for alpha-3/4/5 chains of Collagen IV, which results in defective basement membranes in the kidney, cochlea and eye. The syndrome has different inheritance patterns and historically, was thought of as a disease affecting solely males.

**Case:**

A 15-year-old female presented with pedal oedema, hypertension and proteinuria. She underwent a kidney biopsy which showed findings in keeping with focal segmental glomerulosclerosis. Her condition was refractory to steroids. Steroid-resistant nephrotic syndrome genetics were sent, revealing a rare pathogenic variant in the *COL4A5* gene.

**Conclusion:**

Heterozygous females with X-linked Alport syndrome can develop chronic kidney disease and hearing loss. Clinicians should be mindful when reviewing kidney histology to include Alport syndrome as a differential for female patients. *COL4A3-5* genes should be included in all steroid-resistant nephrotic syndrome genetic panels.

## Case

A 15-year-old female presented with a 2-week history of pedal oedema. She reported no antecedent illness, fever, arthropathy or rash. In early childhood, the patient had attended a general paediatrician for recurrent urinary tract infections. The patient had no significant family history. She had normal growth and development throughout her childhood. Her blood pressure was raised at 140/90 mmHg. Physical examination showed pedal oedema but was otherwise unremarkable. Urinalysis showed 3 + protein and microscopic haematuria. The following laboratory investigations were performed: Complete blood count; coagulation screen; blood urea nitrogen and creatinine; electrolytes; liver function tests; triglycerides, C3; C4; and ANCA. Her creatinine was 45 micromoles per litre, with an estimated glomerular filtration rate of 130 mL/min/1.73 m^2^. She was noted to be anaemic with a haemoglobin of 107 g/dL and a haematocrit of 0.31. Albumin was low at 23 g/L. Urinary protein: creatine ratio was elevated at 474 mg/mmol creatinine. The rest of the results were within normal limits.

**Fig. 1:**
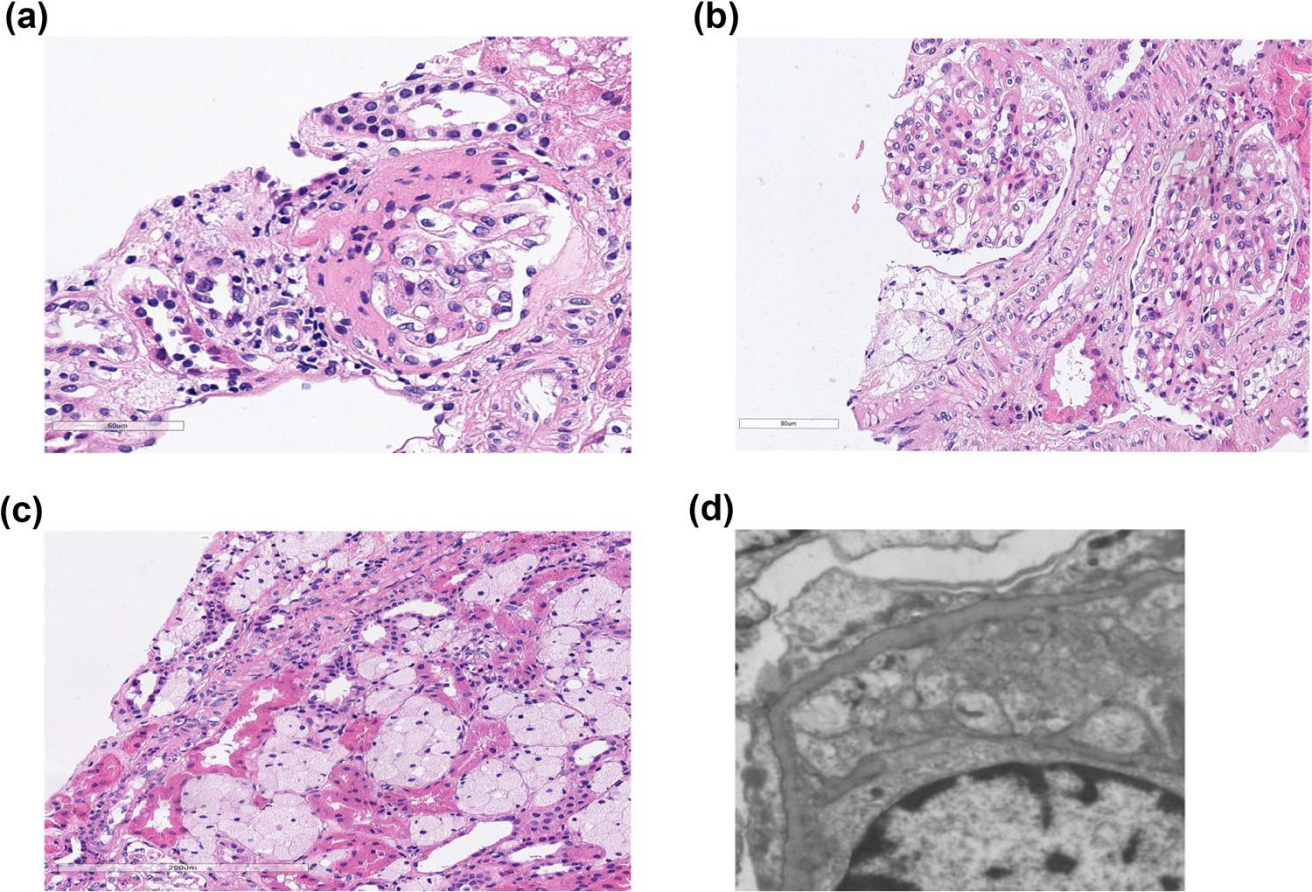
**A**–**D** Kidney histology. **A** Glomerulus showing segmental sclerosis (H&E stained). **B** Tip lesion seen in the right-hand glomerulus (H&E stained). **C** Interstitial foam cells. **D** Electron microscopy

Her initial management included fluid restriction, diuretics and anti-hypertensive therapy. A subsequent kidney ultrasound was performed which was normal. She went on to have a kidney biopsy, prior to the commencement of steroids. She required steroids, diuretics and fluid restriction on discharge. The interval outpatient reviews showed persistence of pedal oedema, heavy proteinuria, hypoalbuminemia and hypertension. After two months of steroid therapy, a genetic panel for steroid-resistant nephrotic syndrome (SRNS) was sent. Her steroid therapy was stopped, and she started regular amlodipine, furosemide and tacrolimus. Her genetic test results revealed a definitive diagnosis. Her immunosuppression was stopped, she remained on diuretics and switched to an angiotensin receptor blocker. Her kidney biopsy histology can be seen in Fig. 1A–D.

## Differential

This adolescent female patient experienced nephrotic-range proteinuria, hypoalbuminaemia, hypertension and haematuria. These findings are consistent with a mixed nephrotic-nephritic picture with a broad differential diagnosis. The differential includes, but is not limited to glomerulosclerosis, post-infectious glomerulonephritis, lupus nephritis and other causes of nephrotic/nephritic syndromes. Complement levels and vasculitis screening yielded no abnormal findings. Viral serology and anti-streptolysin titres were normal, making an infectious aetiology unlikely. Abdominal ultrasound, lactate dehydrogenase and urate were all normal, thus making a malignant cause unlikely. The patient had no recent medication or toxin exposure.

Biopsy showed marked effacement of the epithelial cell foot processes. There was some irregularity to the epithelial side of the basement membrane, but no other structural abnormalities were identified. These findings were in keeping with a diagnosis of focal segmental glomerulosclerosis, and as such, genetic studies for steroid-resistant nephrotic syndrome were performed. Immunofluorescence showed no evidence of an immune complex deposition disease process, thus excluding diagnoses such as IgA nephropathy, C3 glomerulopathy, lupus nephritis, etc. Electron microscopy revealed marked foot process effacement, consistent with a diffuse podocytopathy (Fig. 1A–D).

The selected steroid-resistant nephrotic syndrome genetic panel targeted 69 different genes using a custom designed SureSelect Target Enrichment System kit, with next-generation sequencing using a MiSeq (Illumina) analyser. Genetic studies on the patient revealed a heterozygous, likely pathogenic variant in the *COL4A5* gene – c.3722G > A, p.(Gly1241Asp). At the time of presentation, this variant had not been reported in the literature. The c.3722G > A sequence change is predicted to cause the substitution of a highly conserved glycine residue for an aspartic acid residue at position 1241. Other amino acid changes at the same position have been reported in the literature in association with X-linked Alport syndrome: p.(Gly1241Arg), p(Gly1241Cys), and p.(Gly1241Val) [1, 2]. This was suggestive of a diagnosis of X-linked Alport syndrome. Parental genetics were performed, both of which were negative, suggesting that this variant arose de novo in this patient, supporting its pathogenicity.

Following the diagnosis of X-linked Alport syndrome, the patient’s immunosuppression was stopped and she was commenced on an angiotensin receptor blocker. Given the normal parental genetics, the likelihood of our patient’s siblings being affected was low; however, the risk of gonadal mosaicism could not be fully excluded. As such, urinalysis and blood pressures were taken on all first-degree relatives, all of which were normal. She was transitioned to adult services with normal hearing and eye examinations.

## Discussion

Alport syndrome is a genetically heterogenous disorder resulting from variants in genes coding for alpha-3/4/5 chains of Collagen IV which results in defective basement membranes in the kidney, cochlea and eye. The syndrome has different inheritance patterns: X-linked (COL4A5 mutation), autosomal recessive (homozygous or compound heterozygous mutations in *COL4A3* or *COL4A4* genes), and autosomal dominant (heterozygous mutations in *COL4A3* or *COL4A4* genes). Almost all males affected by X-linked Alport will progress to develop kidney failure, whereas just 12% of females with a heterozygous mutation progress to kidney failure [3].

This case was particularly unusual, given the age at presentation, the severity of her clinical presentation and the histology findings. Deng et al. described a case of an 11-year-old male, accompanied by a literature review, highlighting the importance of keeping Alport syndrome in the differential for both patients with glomerular haematuria and haematuria/proteinuria [4]. Historically, Alport syndrome has been thought of a disease affecting (almost exclusively) men. Alport himself stated that “the females have deafness and haematuria and live to an old age”. Perhaps the most detailed review of X-linked Alport syndrome in females was published by Michelle Rheault in 2011, outlining the progression of clinical knowledge through time, as well as collating the determinants of disease severity in heterozygous females with X-linked Alport syndrome (XLAS) [5]. Subsequent work by Quinlan and Rheault further details the incidence and likelihood of kidney failure in females with XLAS. Their review describes how ninety-five percent of women with XLAS have microscopic haematuria that may start at birth, with the median age of presentation with proteinuria being 7 years [6].

The main explanation for the variable severity of X-linked Alport syndrome in females is X-chromosome inactivation. Selective inactivation of the normal *COL4A5* gene in kidney cells is thought to be a potential aetiology for severe Alport phenotype in heterozygous XLAS females. One previous paediatric case has been published outlining a female with aggressive early onset kidney failure secondary to this [7]. Ijjima and colleagues described a young female who presented initially at age two years, with nephrotic range proteinuria and haematuria. She went on to have a kidney biopsy at ages 6 and 8, with subsequent development of sensorineural hearing loss at the age of 14 years. Her diagnosis was made retrospectively at the age of 19 years, when she presented with microscopic haematuria, nephrotic-range proteinuria, hypoalbuminaemia and primary amenorrhoea [7].

## Conclusion

Heterozygous females with X-linked Alport syndrome can develop chronic kidney disease and hearing loss. They should be followed by nephrologists in the same way their male counterparts are - surveillance with urinalysis, blood pressure monitoring, ophthalmology and audiology assessments are advisable. Further study into X-chromosome inactivation in tissue samples for Alport syndrome is warranted. Clinicians should be mindful when reviewing kidney histology to include Alport syndrome as a differential for female patients. *COL4A3*, *COL4A4* and *COL4A5* genes should be included in steroid-resistant nephrotic syndrome genetic panels.

## Summary

### What is new?

- We present a rare case of X-linked Alport syndrome from a heterozygous, likely pathogenic variant in the *COL4A5* gene–c.3722G > A, p.(Gly1241Asp).
